# Emergence of Rare Bovine–Human Reassortant DS-1-Like Rotavirus A Strains with G8P[8] Genotype in Human Patients in the Czech Republic

**DOI:** 10.3390/v11111015

**Published:** 2019-11-01

**Authors:** Romana Moutelíková, Pavel Sauer, Monika Dvořáková Heroldová, Veronika Holá, Jana Prodělalová

**Affiliations:** 1 Veterinary Research Institute, 62100 Brno, Czech Republic; prodelalova@vri.cz; 2 Institute of Microbiology, University Hospital Olomouc and Faculty of Medicine, Palacký University, 77900 Olomouc, Czech Republic; pavel.sauer@email.cz; 3 Microbiology Institute of Faculty of Medicine, Masaryk University Brno and University Hospital of St. Anne, 65691 Brno, Czech Republic; monika.heroldova@fnusa.cz (M.D.H.); veronika.hola@fnusa.cz (V.H.)

**Keywords:** rotavirus A, G8, gastroenteritis, bovine–human reassortants, Central Europe

## Abstract

Group A Rotaviruses (RVA) are the leading cause of acute gastroenteritis in children and a major cause of childhood mortality in low-income countries. RVAs are mostly host-specific, but interspecies transmission and reassortment between human and animal RVAs significantly contribute to their genetic diversity. We investigated the VP7 and VP4 genotypes of RVA isolated from 225 stool specimens collected from Czech patients with gastroenteritis during 2016–2019. The most abundant genotypes were G1P[8] (42.7%), G3P[8] (11.1%), G9P[8] (9.8%), G2P[4] (4.4%), G4P[8] (1.3%), G12P[8] (1.3%), and, surprisingly, G8P[8] (9.3%). Sequence analysis of G8P[8] strains revealed the highest nucleotide similarity of all Czech G8 sequences to the G8P[8] rotavirus strains that were isolated in Vietnam in 2014/2015. The whole-genome backbone of the Czech G8 strains was determined with the use of next-generation sequencing as DS-1-like. Phylogenetic analysis of all segments clustered the Czech isolates with RVA strains that were formerly described in Southeast Asia, which had emerged following genetic reassortment between bovine and human RVAs. This is the first time that bovine–human DS-1-like G8P[8] strains were detected at a high rate in human patients in Central Europe. Whether the emergence of this unusual genotype reflects the establishment of a new RVA strain in the population requires the continuous monitoring of rotavirus epidemiology.

## 1. Introduction

Gastrointestinal illnesses due to species A rotavirus (RVA) infections among young children greatly contribute to morbidity and mortality rates in developing countries, especially in Asia and Africa. In EU countries, rotavirus gastroenteritis very rarely results in patient death, however the economic and social burden of the disease is considerable [1,2]. Since the WHO recommended the universal vaccination of infants against RVA in 2009, the burden of the disease significantly decreased in countries with an RVA vaccination scheme [3]. Still, according to recent estimates, RVA is considered to be responsible for 122,000–216,000 deaths globally per year in children under five years of age [4,5].

RVAs are members of the *Reoviridae* family and possess a triple-layered capsid enclosing 11 segments of double-stranded RNA. RVAs are classified into G and P types according to differences of the nucleotide sequences of genes coding viral proteins 9 and 4 (VP9, VP4), respectively [6]. Up to now, at least 36 G genotypes and 51 P genotypes have been described [7]. Theoretically, many G/P combinations are possible within the binary system that is utilized to classify RVA genotypes. However, according to the latest report of the European rotavirus surveillance net (EuroRotaNet), there are six main genotypes, G1P[8], G2P[4], G3P[8], G4P[8], G9P[8], and the recently emerged G12P[8], which have been recognized as the most epidemiologically relevant human RVA strains in Europe [8,9].

More detailed analyses of RVA reassortment events or interspecies evolutionary relationships are possible with the use of the whole-genome classification system. All genes encoding six structural virus proteins (VP1–VP4, VP6, VP7) and five (or six in some RVA strains) nonstructural virus proteins (NSP1–NSP5/6) are sorted into defined genotypes on the basis of percentage identity cut-off values. The schematic nomenclature of the RVA whole genome would be Gx-P[x]-Ix-Rx-Cx-Mx-Ax-Nx-Tx-Ex-Hx, representing the genotypes of VP7-VP4-VP6-VP1-VP2-VP3-NSP1-NSP2-NSP3-NSP4-NSP5/6, respectively [10]. Human RVAs mostly belong to one of the three main genogroups (Wa, DS-1, AU-1), where genotypes for each genome segment (except for VP4) of the reference strains were assigned to genotypes 1 (strain Wa), 2 (strain DS-1), and 3 (strain AU-1) [11]. The most widespread human rotavirus strains belong to the Wa-like genogroup (G1P[8], G3P[8], G4P[8], and G9P[8]) and share a majority of genotypes with porcine rotavirus strains. On the other hand, DS-1-like human RVA strains include G2P[4] genotypes and share the majority of genotypes of other segments with bovine strains. The third minor human AU-1-like genogroup has genomic constellation G3-P[9]-I3-R3-C3-M3-A3-N3-T3-E3-H3 and it is believed to have a close evolutionary relationship with canine and feline rotavirus strains [11].

In industrialized countries, rotavirus genotype G8 infection is common in cattle but rarely occurs in humans; however, G8 strains are highly prevalent among humans in some countries in Africa [12] and were also described in Brazil and Chile [13,14,15]. Over the past five years, reports of G8 rotavirus-strain detection have been increasing. Epidemics due to this possibly zoonotic strain were recently reported in several Asian countries: Thailand in 2013/2014, Japan in 2014, Vietnam in 2015, and South Korea in 2017/2018 [16,17,18,19]. In Europe, the occurrence of G8P[8] RVA strains has been very sporadic. In 2006, it was described in Croatia [20], where the G8 strain was found to be closely related to the African G8 strains. Isolated findings of G8 genotypes were also reported in the United Kingdom, Slovenia, Germany, and Italy [21,22,23,24]. Human G8P[8] RVA strains can possess both the Wa-like and the DS-1-like genomic constellation. Strains circulating in Southeast Asia were mainly of the DS-1-like genetic backbone, while G8P[8] strains that were detected in Croatia were described to be of the Wa-like genetic backbone [20].

In this study, we report the occurrence of G8P[8] RVA strains that were detected for the first time in the Czech Republic during 2016–2019. All RVA strains with a G8 VP7 encoding RNA segment were sequenced and characterized. Four of the G8 RVA strains were also submitted to the whole-genome NGS sequencing to understand their genetic diversity and evolution.

## 2. Materials and Methods 

### 2.1. Sample Collection

Rotavirus screening was carried out with the collection of 1680 stool samples gathered from March 2016 to February 2019. Samples were collected in 6 clinical microbiological laboratories located in 5 towns across the eastern part of the Czech Republic. Of the samples, 598 came from patients with gastroenteritis; the rest (*n* = 1082) were sampled from patients with another primary diagnosis (mostly from oncological patients) and 852 of the samples originated from children under 5 years of age. All 225 RVA-positive samples (13.4%; 225/1680) were G- and P-typed. Out of these, 21 were typed as the G8 genotype (9.3%, 21/225). Further analyses were conducted with 16 of these strains that yielded sequences of reasonable quality.

### 2.2. RNA Extraction and RT-PCR Detection

Stool samples were collected in sterile containers and frozen after collection to −70 °C. Suspension preparation and RNA extraction were performed as described previously [25]. RVA detection was carried out with a commercial RT-qPCR test (Human Rotavirus A, Genesig^®^ Standard Kit, Primerdesign™ Ltd, UK). All positive samples were stored at −70 °C for further genetic analyses. PCR assays for the characterization of G- and P-types of positive RVA samples were accomplished with a OneStep Ahead RT-PCR Kit (Qiagen, Germany) with formerly published primers [26,27]. Sanger sequencing of obtained amplicons (1062 bp for VP7 and 876 bp for VP4) was performed by Eurofins Genomics (Ebersberg, Germany).

### 2.3. Phylogenetic Analysis

All the obtained nucleotide sequences were compared to those of RVA strains that were available on the NCBI website using BLAST (http://www.blast.ncbi.nlm.nih.gov/Blast.cgi). Phylogenetic analysis was performed with MEGA 7 software (http://www.megasoftware.net/home). Dendrograms were prepared with the maximum-likelihood method and evolutionary distances were calculated with the use of the Tamura 3-parameter model [28]. To assess the reliability of constructed phylogenetic trees, the bootstrap test (1000 tree replicates) was used. A bootstrap value of ˃75% indicated satisfactory topology of the phylogenetic tree branches; a bootstrap value of 95–100% is very good. To classify the RVA sequence into the corresponding genotype, the percentage of nucleotide-sequence similarity between Czech RVA strains and RVA sequences deposited in the GenBank was calculated using the p-distances method. The assigned genotypes were verified with the RotaC online tool [29] in accordance with the Rotavirus Classification Working Group (RCWG) guidelines. 

Sequences of the G8 VP7 segments, as well as the corresponding VP4 segments of RVAs that were used in this study, were submitted to the GenBank. Accession numbers are listed in Supplementary Table S1.

### 2.4. Whole-Genome Sequencing

Four G8 strains were selected for whole-genome analysis to retrieve the complete backbone of the rotavirus. For the purpose of whole-genome sequencing on Illumina, a modified protocol of the sample preparation that was described earlier was used [30]. Briefly, 25 mL of the 10% fecal suspension was filtered 3 times. A first filtration step was done using 5.0 μm syringe filters, followed by second and third steps with 0.8 and 0.45 μm Minisart^®^ syringe filters, respectively (Sartorius, Göttingen, Germany). The sample was then concentrated 100× with the use of a disposable ultrafiltration centrifugal device Pierce Protein Concentrator, 30K MWCO (Thermo Scientific, Waltham MA, USA) and subsequently in Vivaspin^®^ centrifugal concentrators for 0.4–2 mL (Sartorius, Göttingen, Germany), which resulted in 0.2 mL of the sample. Next, 4 units of DNase I were added (TopBio, Prague, Czech Republic) and the mixture was incubated 10 min/37 °C. After heat inactivation of the enzyme (10 min/65 °C), 20 units of nuclease S1 (Sigma-Aldrich, St. Louis MO, USA) were added into the mixture to cleave all single-stranded RNA and DNA. After 30 min/37 °C, the reaction was stopped by 2.5 μL of 0.5M EDTA (10 min/75 °C). Extraction of the viral RNA for next-generation sequencing (NGS) was performed with QIAampViral RNA Mini Kit (Qiagen, Hilden, Germany), but without the addition of carrier RNA.

Library preparation, Illumina sequencing, and nucleotide-sequence mapping to the rotavirus reference sequences were performed by a commercial provider (SeqMe, Dobříš, Czech Republic). The average number of 250 bp pair-end reads per sample was 775,000 (461,235–1,815,393 reads) and the percentage of mapped reads ranged between 11% and 83.6%, with an average of 32.7% mapped reads. Nucleotide sequences for each genome segment were assembled into contigs using a high-performance graphical viewer Tablet [31].

Whole-genome sequences of representative G8 strain H366/2017 received GenBank accession numbers MN401289–MN401298 (see Supplementary Table S1).

## 3. Results and Discussion

### 3.1. Study Population

A total of 1680 stool samples, gathered between March 2016 and February 2019 from patients with signs of gastroenteritis as well as from patients with other primary diagnoses, were tested for RVA by RT-qPCR. RVA was successfully detected in 225 samples (13.4%; 225/1680). Positivity rates were similar in all 3 years of testing (33/319, 10.3% RVA-positive samples in 2016–2017; 107/832, 12.9% in 2017–2018; 85/566, 15% in 2018–2019).

### 3.2. Rotavirus Genotypes

The most prevalent detected genotypes were G1P[8] (42.7%), G3P[8] (11.1%), G9P[8] (9.8%), G2P[4] (4.4%), and G4P[8] and G12P[8] (each 1.3%). Apart from these expected genotypes, which are considered to be the most prevalent global strains [32], a substantial percentage (9.3%, 21/225) of rare RVA strains with G8P[8] genotype were detected. Epidemiological-screening results are summed up in Table 1.

### 3.3. Specimens with G8 Genotype

**Figure 1:**
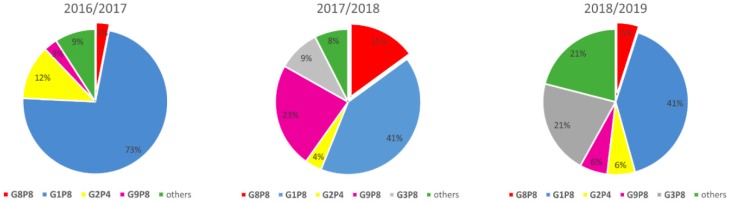
Proportional representation of detected RVA genotypes in human samples during three studied seasons.

The numbers of G8 strains detected throughout the three years of the project varied. The percentage of RVA strains with the G8P[8] genotype is graphically depicted in Figure 1. 

The first detected G8 strain was collected in June 2016 from a 16-month-old girl with acute gastroenteritis (AGE). Next, in 2017, there were 16 G8 samples that were detected in three participating laboratories located in different cities. Out of these, seven patients were under five years of age (two of them with febrile convulsions, others with AGE), two other patients were under 15 years of age (both with AGE), and the seven remaining adult patients were primarily hospitalized with different disorders (cardiological or oncological diagnosis); their RVA infection was most likely of nosocomial origin. In 17 G8 strains, both G- and P-types were determined. Comparison of the VP7 and VP4 gene sequences of those G8P[8] strains showed that the mean nucleotide-sequence identity of VP7 genes was 99.8%, with a range of 99.4–100%, and that of VP4 genes was 99.4%, with a range of 98.6–100%. These results led us to the hypothesis that the G8P[8] strains originated from a single source.

### 3.4. Phylogenetic Analysis of VP7 Segment

**Figure 2:**
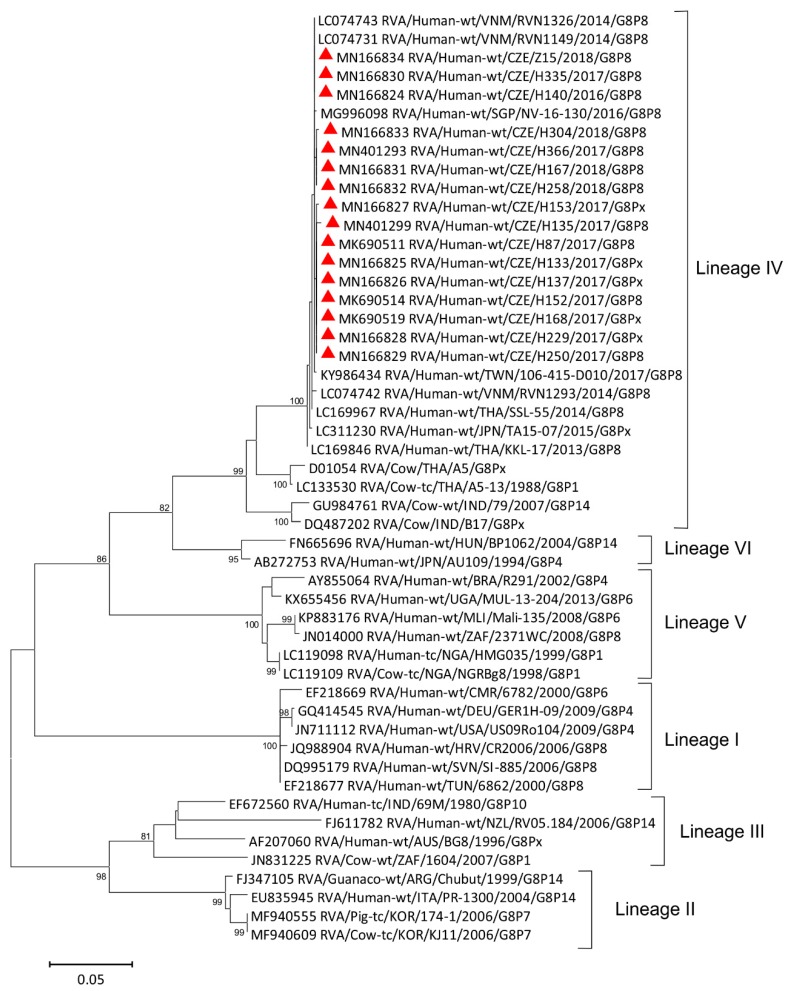
Phylogenetic analysis of the VP7 gene of G8 rotavirus strains used in the phylogenetic study of RVA strains. The red triangle indicates G8 RVA strains detected in the Czech Republic. The Tamura 3-parameter model was used for a maximum-likelihood method. Bootstrap values higher than 75% are shown at branch nodes. The scale bar indicates nucleotide substitutions per site.

Phylogenetic analysis of G8 strains detected in the Czech Republic, together with human and animal G8 strains available in the GenBank, is displayed in Figure 2.

The Czech strains clustered exclusively with bovine-like human strains formerly described in Vietnam [18], Singapore [33], Thailand, and Japan [16,17]. The nucleotide similarities of the Czech G8 sequences and the Vietnamese strains were 99.7–100%. Rare RVA strains of the G8 genotype that were isolated in other European countries like Germany, Slovenia, and Croatia (GER-H-09, Sl-885, CR2006, respectively) formed a distant cluster, previously determined as lineage I according to the work of Agbemabiese et al. [34]. Another European G8 strain, detected in Hungary in 2004 [35], was clustered into a distinct lineage together with the Japanese G8 strain. Nucleotide similarities of these European G8 strains and Czech G8 strains were very low—they ranged between 77.8% (CZE/H304/2018 and GER-H-09 strains) and 86.8% (CZE/H140/2018 together with CZE/Z15/2018 and HUN/BP1062). It is of interest that Czech, Vietnamese, Thai, and other Southeast Asian G8 strains, which were tightly clustered in a lineage IV, shared nucleotide similarity lower than 80% with all strains from lineage I, comprising mostly G8 strains that originated in Africa. This evidences the independent evolution of G8 strains in Africa and Asia. The majority of G8 strains that are found in human samples may be reassortants between animal and human rotaviruses; they contain porcine rotavirus-like genes inserted in their Wa-like genome, or bovine rotavirus-like genes on the DS1-like genomic configuration [36,37].

The Czech strains that were analyzed in this study showed high similarity (92.5–94.5%) of nucleotide sequence in the VP7 segment with bovine RVA strains that were described in India (strain 79/2007) or Thailand (A5-13/1988) [38,39]. It was earlier hypothesized that the G8 VP7 gene of the Southeast Asian bovine-like human G8 strains is the result of a recent transfer of the bovine VP7 gene to a human strain. Hoa-Tran et al. validated this hypothesis by BEAST analysis and estimated that the transfer of the bovine G8 VP7 gene to a human strain occurred between 2007 and 2012 [18].

### 3.5. Whole-Genome Sequencing

Whole-genome-based analysis is a reliable method for obtaining conclusive data on the origin of an RVA strain and tracing its evolutionary pattern. The NGS sequencing of four Czech G8 strains of RVA yielded enough quality sequence data to be able to retrieve whole-genome sequences. As nucleotide similarities of the more conserved segments (VP1-VP3, VP6, NSP1–NSP5) were close to identity (99% in VP6, 99.5–100% in other genomic segments), only one whole-genome sequence of representative strain H366/2017 was entered into the GenBank. The constellation of all sequenced RVA strains was DS-1-like (G8-P[8]-I2-R2-C2-M2-A2-N2-T2-E2-H2).

### 3.6. Phylogenetic Whole-Genome Analysis 

**Figure 3:**
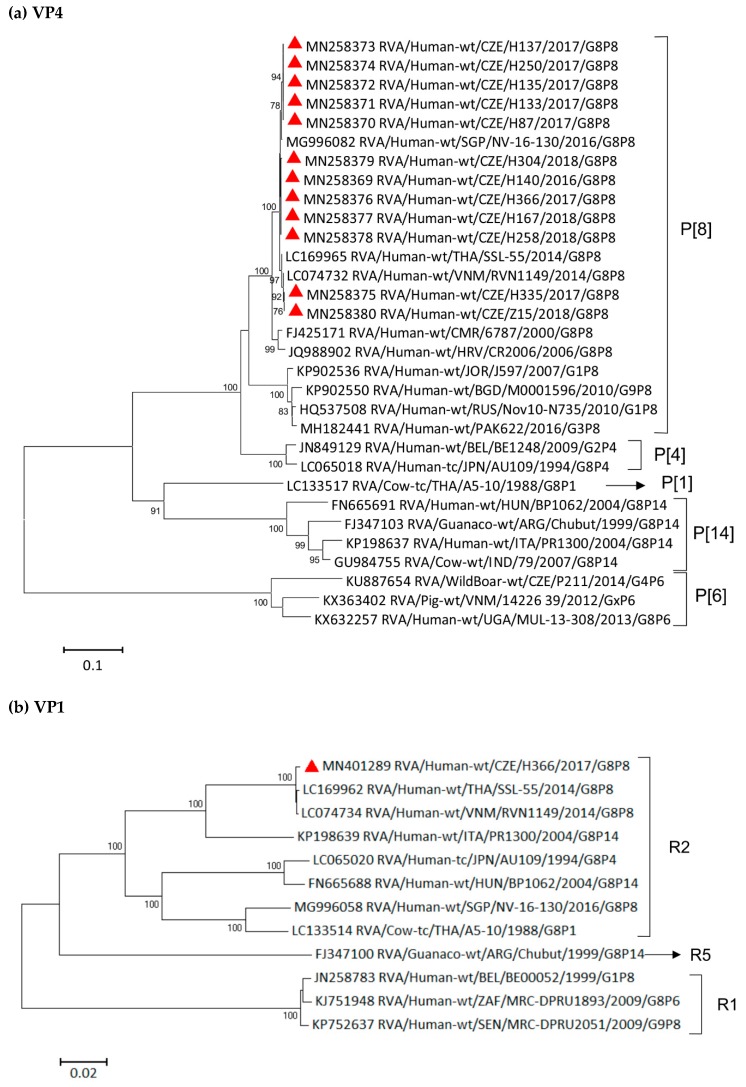

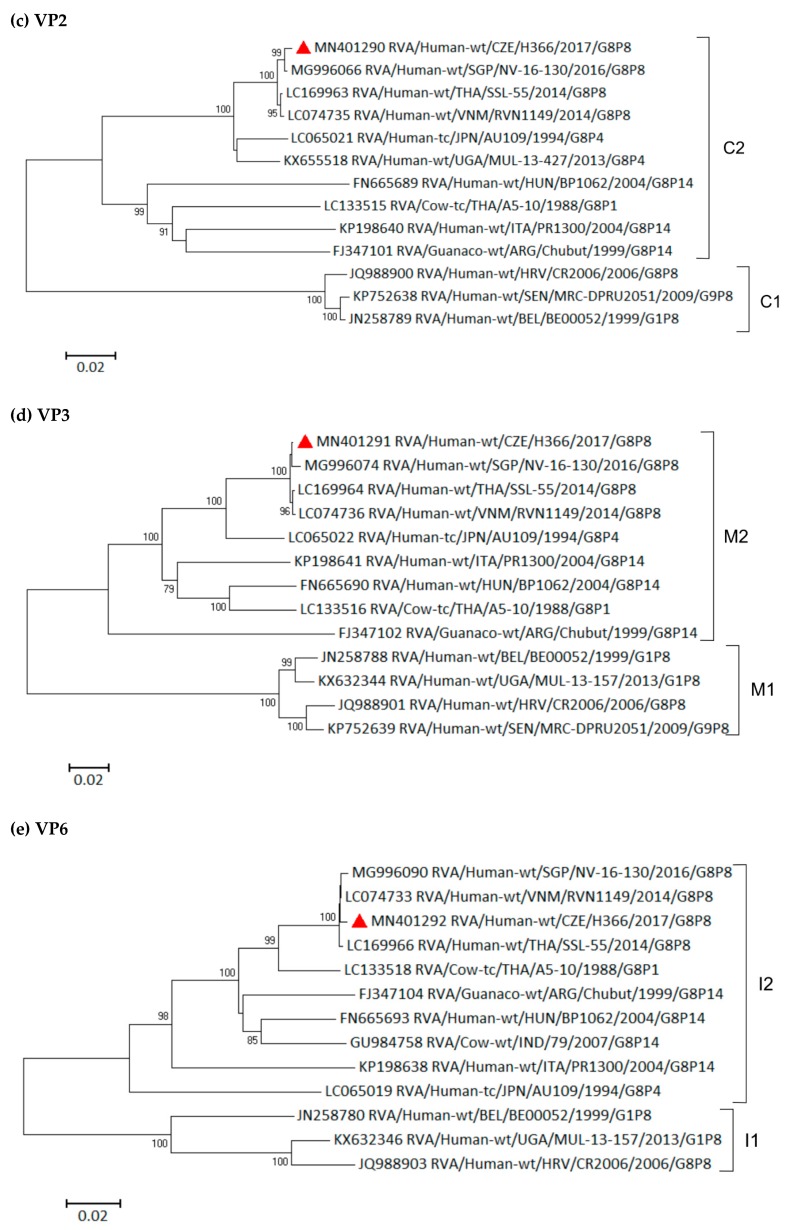

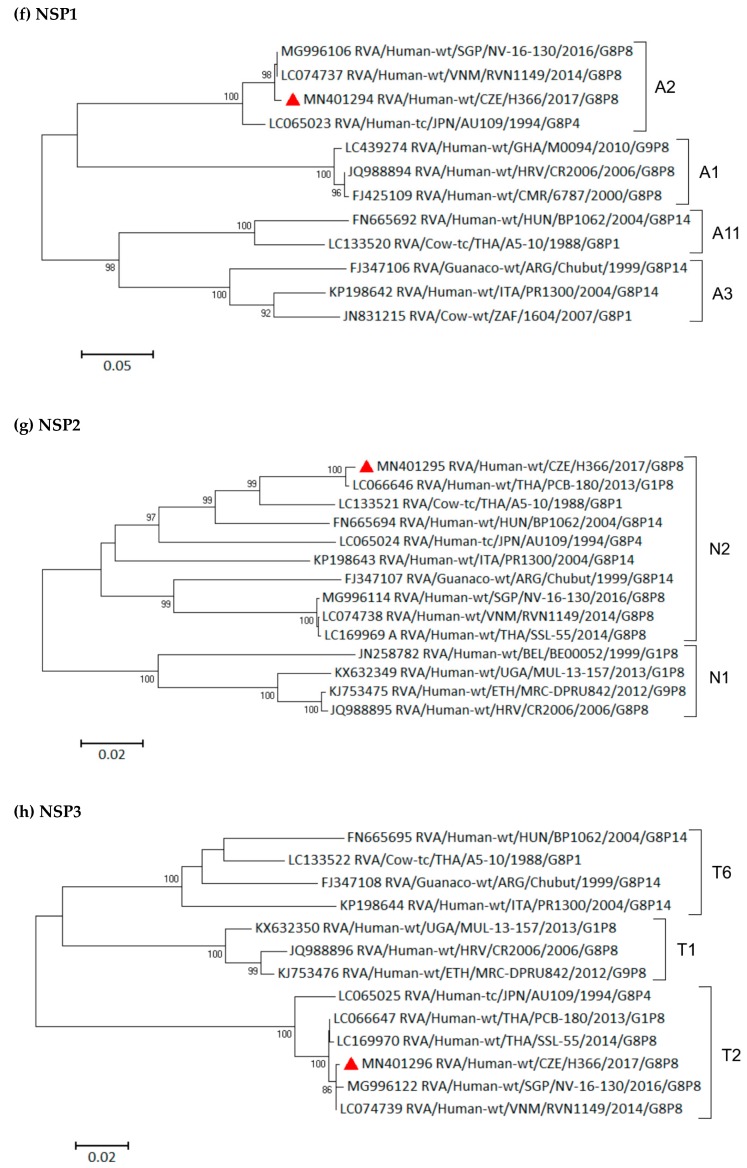

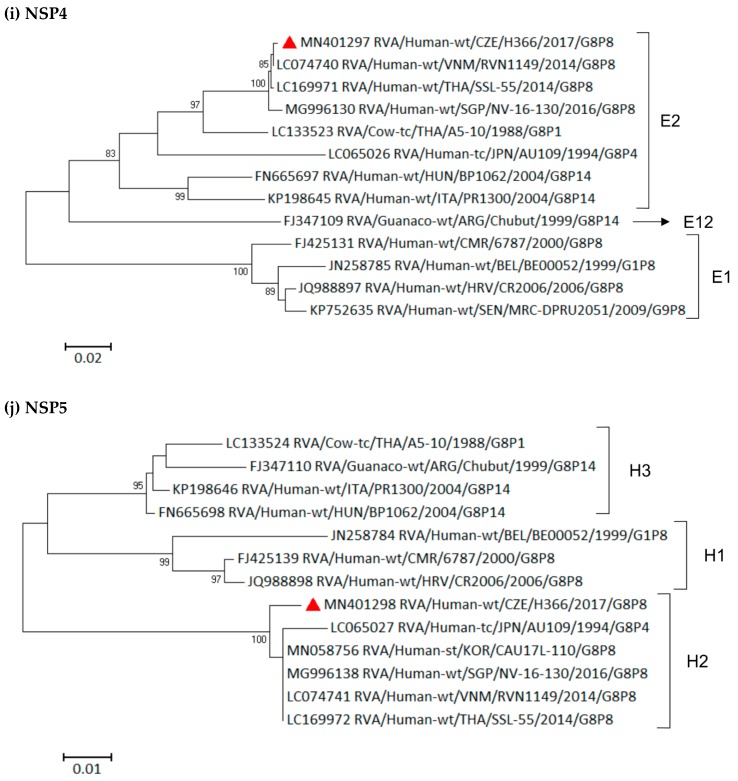
Phylogenetic analysis of the (**a**) VP4, (**b**) VP1, (**c**) VP2, (**d**) VP3, (**e**) VP6, (**f**) NSP1, (**g**) NSP2, (**h**) NSP3, (**i**) NSP4, (**j**) NSP5 genes of G8 rotavirus strains used in the phylogenetic study and other cognate RVA strains available in the GenBank. The red triangle indicates G8 RVA strains detected in the Czech Republic. The Tamura 3-parameter model was used for a maximum-likelihood method. Bootstrap values higher than 75% are shown at branch nodes. The scale bar indicates nucleotide substitutions per site.

Initial comparisons of the 10 remaining genomic segments (encoding VP1–VP4, VP6, NSP1–NSP5) of representative Czech strain H366/2017 with the corresponding genes of other completely sequenced RV strains found in the GenBank showed the highest nucleotide-sequence similarities with Vietnamese DS-1-like G8P[8] strain RVN1149 or Thai strain SSL-55 in most of the analyzed genes (Figure 3a–j). Similarities of ≥99% were observed in VP4 (99.0–99.9%), VP1 (99.7%), VP2 (99.2%), VP3 (99.7%), VP6 (99.7%), NSP1 (99.4%), NSP3 (99.9%), NSP4 (99.8%), and NSP5 (99.1%).

The constructed phylogenetic trees showed that the only evolutionarily more distant genomic segment was NSP2. Although it still belongs to the same N2 subcluster, it only shares 86.8% and 86.9% similarity with RVN1149 and SSL-55, respectively. A close evolutionary relationship in the NSP2 segment was observed between the Czech strain H366/2017 and G1P[8] strains described during the gastroenteritis outbreak in children in Thailand [40]. Those intergenogroup reassortant Thai RVA strains contained a Wa-like G/P genotype on a DS-1-like backbone and, in NSP2, shared 99.6% nucleotide similarity with the Czech strain. As the reassortant G1P[8] outbreak in Thailand coincided with the G8P[8] outbreak in Vietnam (2012–2015), we may assume that prior to this period, many reassortment events (between human and bovine strains resulting in DS-1-like G8P[8] strains or between human Wa-like and DS-l-like strains) occurred.

### 3.7. Spread of G8P[8] Rotavirus Strains

The route of G8P[8] strains that were spread into Central Europe is hard to establish. However, the Vietnamese community (estimated 100,000 people) in the Czech Republic is the third most numerous in Europe (after France and Germany) and is the biggest when considered as percentage of the population (reaching 1% of inhabitants) [41]. With the globalization of trade and frequent travel of EU citizens, either for business or for leisure, we could speculate about direct import from one of the countries in Southeast Asia.

## 4. Conclusions

In this report, human–bovine reassortant RVA strains of the G8P[8] genotype were detected as the cause of an outbreak of acute gastroenteritis in human patients in Central Europe. The high prevalence of DS-1-like G8P[8] strains that were described in this study indicates that these strains are well-adapted to human–human transmission in an industrialized country. Therefore, it is very likely that these G8P[8] rotavirus strains could spread to other regions in the near future. Continuous surveillance of the genotypes of RVA isolates is recommended in order to monitor circulating wild-type strains, as well as rotavirus genotype constellations, to understand rotavirus diversity and their evolutionary patterns.

## Figures and Tables

**Table 1:** Combinations of G and P types characterized in Group A Rotaviruses (RVA)-positive stool samples during 2016–2019.

	Strain	2016–2017	2017–2018	2018–2019	Total
Fully typed strains	G1P[8]	24	39	33	96
	G2P[4]	4	2	4	10
	G2P[8]	-	1	-	1
	G3P[8]	-	10	15	25
	G3P[9]	1	-	1	2
	G4P[8]	-	-	3	3
	G8P[8]	1	12	4	17
	G9P[4]	-	-	2	2
	G9P[8]	1	16	5	22
	G12P[8]	-	2	1	3
Partially typed strains	G1P[nd]	-	5	-	5
	G2P[nd]	1	2	1	4
	G3P[nd]	-	-	2	2
	G8P[nd]	-	4	-	4
	G9P[nd]	-	9	-	9
	GndP[4]	-	-	2	2
	GndP[8]	1	3	8	12
	GndP[nd]	-	1	4	5
Mixed infections	G9P[4][8]	-	1	-	1
Total		33	107	85	225

Abbreviations: nd, not determined; -, not detected.

## Supplementary Materials

The following are available online at https://www.mdpi.com/1999-4915/11/11/1015/s1, Table S1: GenBank accession numbers of Czech rotavirus strains with G8 genotype.
