# Supplementary material for: Emergence of Rare Bovine–Human Reassortant DS-1-Like Rotavirus A Strains with G8P[8] Genotype in Human Patients in the Czech Republic
Source: Viruses. 2019 Nov 1;11(11):1015. doi: 10.3390/v11111015 (PMC6893433; doi:10.3390/v11111015)
Supplement: Supplementary file 1 [file viruses-11-01015-s001.pdf]

Supplementary table S1. GenBank accession numbers of Czech rotavirus strains with G8 genotype.

Strain	VP7 segment	VP4 segment	VP1-VP3, VP6, NSP1-NSP5 segments
H140/2016	MN166824	MN258369	-
H87/2017	MK690511	MN258370	-
H133/2017	MN166825	MN258371	-
H135/2017	MN401299	MN258372	-
H137/2017	MN166826	MN258373	-
H152/2017	MK690514	-	-
H153/2017	MN166827	-	-
H168/2017	MK690519	-	-
H229/2017	MN166828	-	-
H250/2017	MN166829	MN258374	-
H335/2017	MN166830	MN258375	-
H366/2017	MN401293	MN258376	MN401289- MN401291, MN401292, MN401294- MN401298
H167/2018	MN166831	MN258377	-
H258/2018	MN166832	MN258378	-
H304/2018	MN166833	MN258379	-
Z15/2018	MN166834	MN258380	-
